# Biofilm and Gene Expression Characteristics of the Carbapenem-Resistant Enterobacterales, *Escherichia coli* IMP, and *Klebsiella pneumoniae* NDM-1 Associated with Common Bacterial Infections

**DOI:** 10.3390/ijerph19084788

**Published:** 2022-04-14

**Authors:** Majid Al-Bayati, Shivanthi Samarasinghe

**Affiliations:** Leicester School Allied Health Sciences, Faculty of Health & Life Sciences, De Montfort University, Leicester LE1 9BH, UK; majid.al-bayati@my365.dmu.ac.uk

**Keywords:** carbapenems-resistant bacteria, *E. coli*, *K. pneumonia*, growth conditions, biofilm assay, confocal scanning laser microscopy, qPCR biofilm-related gene expression, *Enterobacteriaceae*

## Abstract

In light of the limited therapeutic options with Carbapenem-Resistant Enterobacterales (CRE) infections, understanding the bacterial risk factors, such as biofilm formation and related gene expression of CRE, is vital. This study investigates the biofilm formation and biofilm-related gene expression of two enteric Enterobacterales with major CR determinants *Escherichia coli* IMP and *Klebsiella pneumoniae* NDM-1, which were seen in high prevalence in most common bacterial infections over the past few years. To our knowledge, this is the first study that demonstrated the relationship between biofilm formation and the related gene expression, to understand the potential molecular mechanisms during the biofilm formation in CRE. Biofilms were quantified by tissue culture plate assay at the stages of the biofilm development: initial attachment (6 h), microcolony formation (12 h), maturation (24 h), and dispersion (48 h). In a dispersion, event bacteria detach without any mechanical means and colonise another area. To investigate the influence of different growth conditions on biofilm formation, biofilms were quantified under different growth conditions. In parallel, quantitative real-time PCR (qPCR) assessed the biofilm-related gene expression of a cluster of genes, including biofilm maturation, quorum sensing, stress survival, and antibiotic resistance. Structural changes during biofilm development were assessed via confocal laser scanning microscopy (CLSM). We observed that the biofilm formation of CRE is correlated with the biofilm development stages, with maximum biofilm observed at 24 h at the maturation stage. Our data also showed that biofilm growth, under the condition tested, is the major factor influencing the variability of biofilm gene expression quantification assays. qPCR analyses have demonstrated that the expression of biofilm-related genes is highly correlated with phenotypic biofilm development, and these findings can be further expanded to understand the variation in regulation of such genes in these significant CRE pathogens. Our study demonstrated that both CRE strains, *E. coli* IMP and *K. pneumoniae* NDM-1, are high biofilm formers, and genes involved in biofilm development are upregulated during biofilm growth. The characteristic of the increased biofilm formation with the upregulation of antibiotic-resistant and biofilm-related genes indicates the successful pathogenic role of biofilms of these selected CRE and is attributed to their multi-drug resistance ability and successful dissemination of CRE in common bacterial infections.

## 1. Introduction

The Enterobacterales, such as *Escherichia coli* and *Klebsiella pneumoniae*, are the most common causes of community and healthcare-associated urinary tract infections and are also associated with the most lethal lower respiratory and bloodstream infections [1,2,3]. There has been a vicious cycle of increasing resistance in Enterobacterales, specifically with *E. coli* and *K. pneumoniae*. Global dissemination of extended-spectrum beta-lactamases (ESBLs) among Enterobacterales has resulted in increased resistance to all penicillin and cephalosporin antibiotics, which has led to an increase in consumption of carbapenem [4]. Carbapenem has been recognised as a last-resort drug, as well as often being the treatment choice for infections caused by multidrug-resistant Enterobacterales [5]. However, the increased consumption of carbapenem has, in turn, increased selection pressure and facilitated the global spread of carbapenem-resistant Enterobacterales (CRE) [6]. Infections caused by CRE are a major health concern due to the limited therapeutic options and high mortality rates associated with these infections [7]. According to the published literature, there are three major classes of carbapenem-resistant (CR) determinants. They are classified as Class A, *Klebsiella pneumoniae* carbapenemase (KPC); Class B, Metallo-β-lactamases (MBLs), such as New Delhi MBL (NDM), Verona integrin-encoded MBL (VIM), and imipenemase (IMP); and Class D, oxacillinases (OXA)-type enzymes such as OXA-48-like carbapenemases [7,8]. It is reported that genes encoding for these CR determinants are mainly associated with mobile genetic elements enabling their rapid dissemination [7,8]. Virulence factors of CRE include different adhesins, haemolysin production, serum resistance, and biofilm formation [5]. A biofilm is a structured community of bacterial cells that are enclosed in a self-produced matrix and adherent to an inert or living surface [9]. Bacterial biofilms are recognised as an important cause of many infections as 65–80% of all bacterial infections are related to biofilm formation [10,11,12]. Studies have shown that specific physiological growth conditions and genetic interactions within the biofilms cause a dramatic increase in intolerance to the antimicrobial agents, as well as the association between antibiotic resistance and biofilm formation [13,14,15].

Although some surveys have shown that different growth conditions influence biofilm formation and, in turn, the association with antibiotic resistance [14], how the growth conditions affect the biofilm-related-gene expression was not well elucidated in CRE. Specifically, how the growth condition influences the expression of the genes responsible for the bacterial cell communication pathways in CRE. One form of bacterial cell to cell communication is known as quorum sensing (QS). QS is a cell density-dependent chemical signalling system that controls the gene expression relaying on the cell density and regulates bacterial virulence, including that of motility, adhesion, and biofilm formation [16,17].

The QS system for Gram-negatives includes the autoinducer-2 (AI-2) system, where the gene products of *luxS* are collectively referred to as AI-2 molecules [18]. In *E. coli* and *K. pneumoniae*, some genes have been associated with the AI-2 system, and it has been shown that biofilm reduction and virulence gene expression can be attributed to the repression of AI-2 QS systems [19]. Furthermore, it has been shown that *luxS* produces AI-2 molecules under low nutrient conditions that are exported and can signal other cells to form a biofilm [18]. Hence, it is essential to evaluate how physiological/growth conditions influence biofilm development and the gene expression profiles, and in turn how these characteristics are correlated with the biofilm-associated drug-resistant pathogens.

The increasing incidence of CRE is a serious threat due to the associated poor clinical outcomes, and CRE outbreaks have already been reported worldwide [20,21,22]. This burden is exacerbated when CRE strains produce biofilms as they cause increased resistance to antimicrobial agents [13]. Therefore, this study aims to understand the relationship between the increased resistance of CRE isolates, *E. coli* IMP and *K. pneumoniae* NDM-1, with the biofilm formation and genes encoding for antibiotic-resistant and biofilm-related gene expressions under different growth conditions.

## 2. Materials and Methods

### 2.1. Bacterial Strains and Growth Conditions

Carbapenem-resistant *E. coli* IMP (NCTC-13476), *K. pneumoniae* NMD-1 (NCTC-13443), and the non-resistant control strains *Escherichia coli* (NCTC-12241), and *Klebsiella pneumoniae* (NCTC-9633), from the Public Health England (PHE)-typed culture collection were used in this study. Previous studies on biofilm analysis showed that there is a heterogeneity of biofilm formation in different media in a given strain [23,24].

To assess the effect of different growth media on biofilm development, we considered two types of growth media. Tested strains were cultured in two types of media, a nutritionally poor medium, Minimal Salts Medium (AB), and nutrient-rich Luria–Bertani Broth (LB). AB medium was used to mimic the low nutrient condition. For biofilm analysis collectively used three passages for each tested strain. Biofilms were quantified at different growth stages of biofilm development such as initial attachment (6 h), microcolony formation (12 h), maturation (24 h), and dispersion (48 h), at 37 °C under static and shaker conditions. The effect of aeration and distribution of nutrients on the biofilm formation process were also explored in this study by incubating test strains under shaking and static conditions.

### 2.2. Quantification of Biofilms Using the 96-Well Tissue Culture Plate Assay

*E. coli* IMP and *K. pneumoniae* NMD-1 biofilms were analysed using the 96-well tissue culture plate assay, according to the previously described methods with some modifications [18,25]. In summary, the strains were grown overnight in AB and LB media (5 mL) at 37 °C, where the optical density of each inoculum was adjusted to OD_595_, 0.08 nm, where OD_595_ is the optical density measured at 595 nm. A 200 µL of inoculated cell suspension was then added to four 96-well plates and incubated at 37 °C in static and shaking conditions at 70 rpm, where the incubation was conducted for various time intervals of 6, 12, 24, and 48 h. After the intended period of incubation, the broth was eliminated from the plate wells using Pasteur pipettes, and the wells were rinsed twice with sterile phosphate-buffered saline (PBS) to remove any non-adherent planktonic cells. The biofilms in the wells were then stained by the addition of 125 µL of 1% crystal violet and incubated for 30 min at room temperature. The stain was rinsed with sterile water until clean and the dye was re-solubilised with 33% *v*/*v* acetic acid [26], which was then left at room temperature for 30 min. Finally, the optical density of each well was measured at OD_595_ (measured at 595 nm) to evaluate the amount of biofilm formed. A medium-only negative control was included in each assay. The biofilms were classified in categories as strongly adherent (OD_595_ > 0.5), moderately adherent (OD_595_ 0.3–0.5), weakly adherent (OD_595_ < 0.3), lack of biofilm OD_595_ < 0.15 and non-adherent (OD_595_ = 0). All the tests for each microorganism were performed in triplicate at each growth stage, under shaking or static conditions, and for each growth media [27].

### 2.3. Biofilm Analysis Using Confocal Laser Scanning Microscopy (CLSM)

CSLM analysis was performed based on previously established protocols [25,26,27,28,29] *E. coli* IMP and *K. pneumoniae* NDM-1 were grown overnight in LB media (5 mL) at 37 °C, where the optical density of each inoculum was adjusted to OD_595_, nm 0.08, and 700 µL aliquots were placed into on µ-slide two wells with a chambered coverslip, (Ibidi, Gräfelfing, Germany). Each well with a lid was incubated statically at 37 °C for different periods (6, 12, 24, and 48 h). After incubation, the µ-slide was carefully washed with PBS buffer to remove all planktonic cells. An amount of 6 µL of Live/Dead staining (*LIVE/DEAD* BacLight^TM^ Bacterial Viability Kits, Molecular Probes, Bleiswijk, The Netherlands) was added according to the manufacturer’s instructions at room temperature and set for 15 min in the dark. The biofilms were fixed using 500 µL of Image- It fixative solution of 4% Formaldehyde (Thermo Scientific, Horsham, UK) and incubated at room temperature in the dark. The biofilms on the wells were washed with PBS three times and ProLong^TM^ Gold Antifade reagents (Molecular Probes, Bleiswijk, The Netherlands). The test samples were then imaged via confocal laser scanning microscopy using an Olympus FV 1000 laser scanning microscope (Olympus, Tokyo, Japan). All the tests for each microorganism were performed in duplicate and representative images were selected for each growth condition.

### 2.4. RNA Isolation from Bacterial Biofilms and cDNA Synthesis

Bacterial strains were inoculated overnight in AB and LB media (5 mL) at 37 °C, where the optical density of each inoculum was adjusted to OD_595_, 0.08 nm, and 6 mL aliquots were transferred into the six-wells of a polystyrene tissue culture plate. The plate was covered with a lid and then incubated statically for 6, 12, 24, and 48 h at 37 °C. After incubation, the wells were carefully washed with sterile PBS buffer. The adhering bacterial cells in each well were disrupted and transferred into a cold sterile PBS buffer. The suspension was then treated with a double volume of RNA protection reagent (QIAGEN, Hilden, Germany) and immediately incubated for 5 min at room temperature and vortexed for 5 s. Cell pellets were obtained by centrifuging at 10,000× *g* for 15 min, then resuspended in 100 µL of RNase-free water (QIAGEN, Germany), after which the cell pellets were stored at −80 °C for subsequent RNA isolation [30].

Bacterial pellets were suspended in 100 μL of RNase-free water (QIAGEN, Hilden, Germany). The RNA isolation was performed using a RNeasy^®^ Power Biofilm Kit (QIAGEN, Hilden, Germany), according to the manufacturer’s guidance [31], whilst DNA decontamination treatment was performed using Turbo DNase (QIAGEN, Hilden, Germany). The RNA was quantified by measuring the absorbance at 260–280 nm using a Nanodrop spectrophotometer ND (Thermo Fisher Scientific, Waltham, MA, USA), and the RNA quality was determined via agarose gel electrophoresis. The RNA was reverse transcribed to cDNA using RevertAid H Minue First Stand cDNA Synthesis (Thermo Fisher Scientific, Waltham, MA, USA) according to the manufacturer’s protocol.

### 2.5. Primers and Their Specificities for qPCR

**Figure 1:**
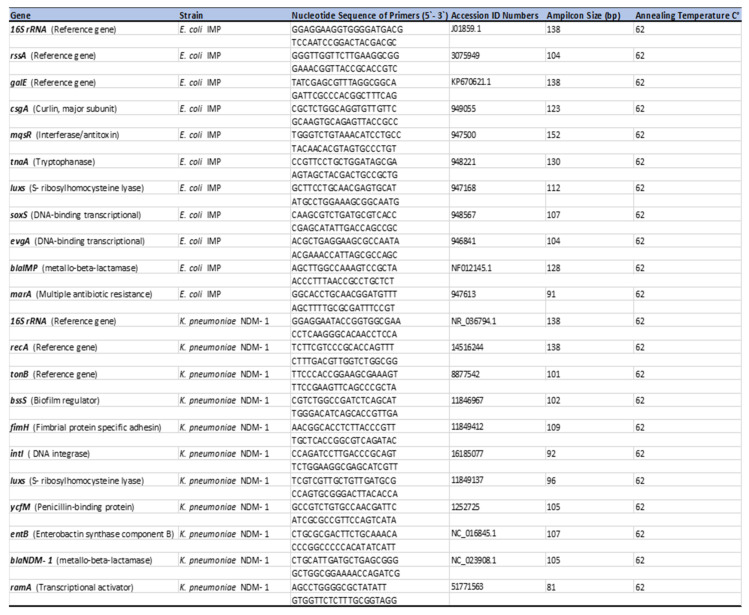
A list of all the genes examined with their relevant function. Indicates corresponding primer sequences for each gene in *Escherichia coli* IMP and *K. pneumoniae* NDM-1. Optimised PCR annealing temperature for all the gens at 62 °C.

The NCBI database www.ncbi.nim.nih.gov (accessed on 1 December 2019) was used to select gene-specific targets, and primers were designed using the Integrated DNA Technologies, Coralville, IA, USA (IDT) website tools www.idtdna.com (accessed on 1 December 2019). Primers were purchased from IDT. The primers used for *E. coli* IMP and *K. pneumoniae* NDM-1 are listed in (Figure 1). Annealing temperatures were optimised for each primer pair using the melting curve analysis and post-PCR agarose gel electrophoresis for the PCR products obtained. Primer efficiencies were calculated using serial dilutions of cDNA (diluted for 100 ng/µL, 50 ng/µL, 10 ng/µL, 1 ng/µL, and 0.1 ng/µL).

### 2.6. Quantitative Real-Time PCR (qPCR)

The transcript levels of the eight genes in *E. coli* IMP and eight genes in *K. pneumoniae* NDM-1 which were responsible for antibiotic resistance and biofilm-related activity measured at four different growth stages (i.e., after 6, 12, 24, and 48 h of incubation) and in AB and LB media via qPCR. The strains were grown overnight in AB and LB media (5 mL) at 37 °C, where the optical density of each inoculum was adjusted to OD_595_, 0.08 nm. For each strain, selected genes were categorised into biofilm formation, quorum sensing, stress survival, and antibiotic resistance. The qPCR reaction was carried out using a SensiFAST SYBR NO-ROX Kit (BioLine, Memphis, TN, USA) and according to the previously published study [30]. The total reaction volume was set at 20 μL per sample and contained 200 ng/mL cDNA and 400 nM of each primer. All reactions were performed as technical triplicates using 96-well plates and on two biological replicates.

### 2.7. qPCR Data Analysis Using Modified ∆∆Ct Method

The expression of the eight selected genes in *E. coli* IMP and eight selected genes in *K. pneumoniae* NDM-1 at 12, 24, and 48 h was calculated relative to the sample at 6 h (calibrator sample) and normalised to the expression of the reference gene (*16S rRNA* for each organism). A calibrator sample was selected at 6 h, as at this time a minimal amount of biofilm formation was observed compared to 12, 24, and 48 h, where relatively high biofilm formation was observed [30]. The X-fold change of the transcription level was calculated using the modified ΔΔCt method [32], with the modification of primer efficiencies that were calculated utilising the serial dilutions of cDNA (100 ng/μL, 50 ng/μL, 10 ng/μL, 1 ng/μL, and 0.1 ng/μL), were included in subsequent ΔΔCt calculations. The expression fold change of a target gene = 2^ΔΔCq (where ΔCq = Cq [target gene] − Cq [reference gene], and ΔΔCq = ΔCq [test] − ΔCq [calibrator]).

### 2.8. Statistical Analysis

**Biofilm analysis:** The one-way ANOVA statistical analysis method was used to determine the statistically significant differences between the means of biofilm-forming capacity at 6 h, 12 h, 24 h, and 48 h group under the given media (AB media or LB media) and in a given condition (static or shaking) in a given strain (*E. coli* IMP or *K. pneumoniae* NDM-1). Prism 6 version 6.01 and Microsoft Excel 365 were used to perform statistical calculations. A *p*-value < 0.05 is considered a statistically significant maximum biofilm formation. The analysis was performed on three biological replicates and the representative values are the means of triplicates. Collectively, three passages were used for each tested strain.

**Gene expression analysis:** The one-way ANOVA statistical analysis method was also used to determine the statistically significant differences in relative gene expression of a target gene between 12 h, 24 h, and 48 h groups under the given media (AB media or the LB media) and in a static condition in a test strain (*E. coli* IMP or *K. pneumoniae* NDM-1). A *p*-value < 0.05 is considered a statistically significant increased fold expression, and when *p*-value > 0.05 is considered a statistically significant decreased fold expression value. The analysis was performed on two biological replicates and the representative values are the means of triplicates.

## 3. Results

### 3.1. Biofilm Quantitative Assay

**Figure 2:**
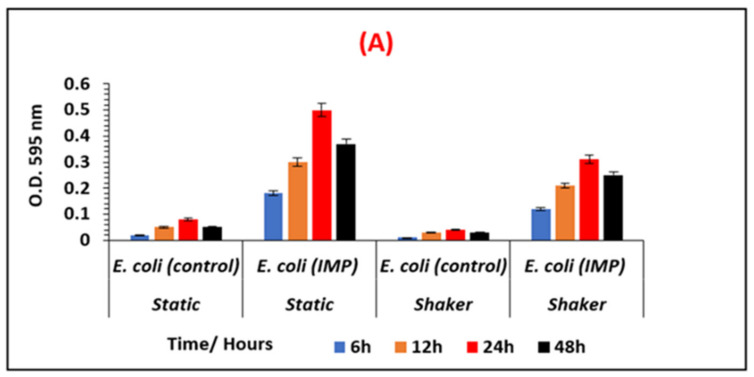

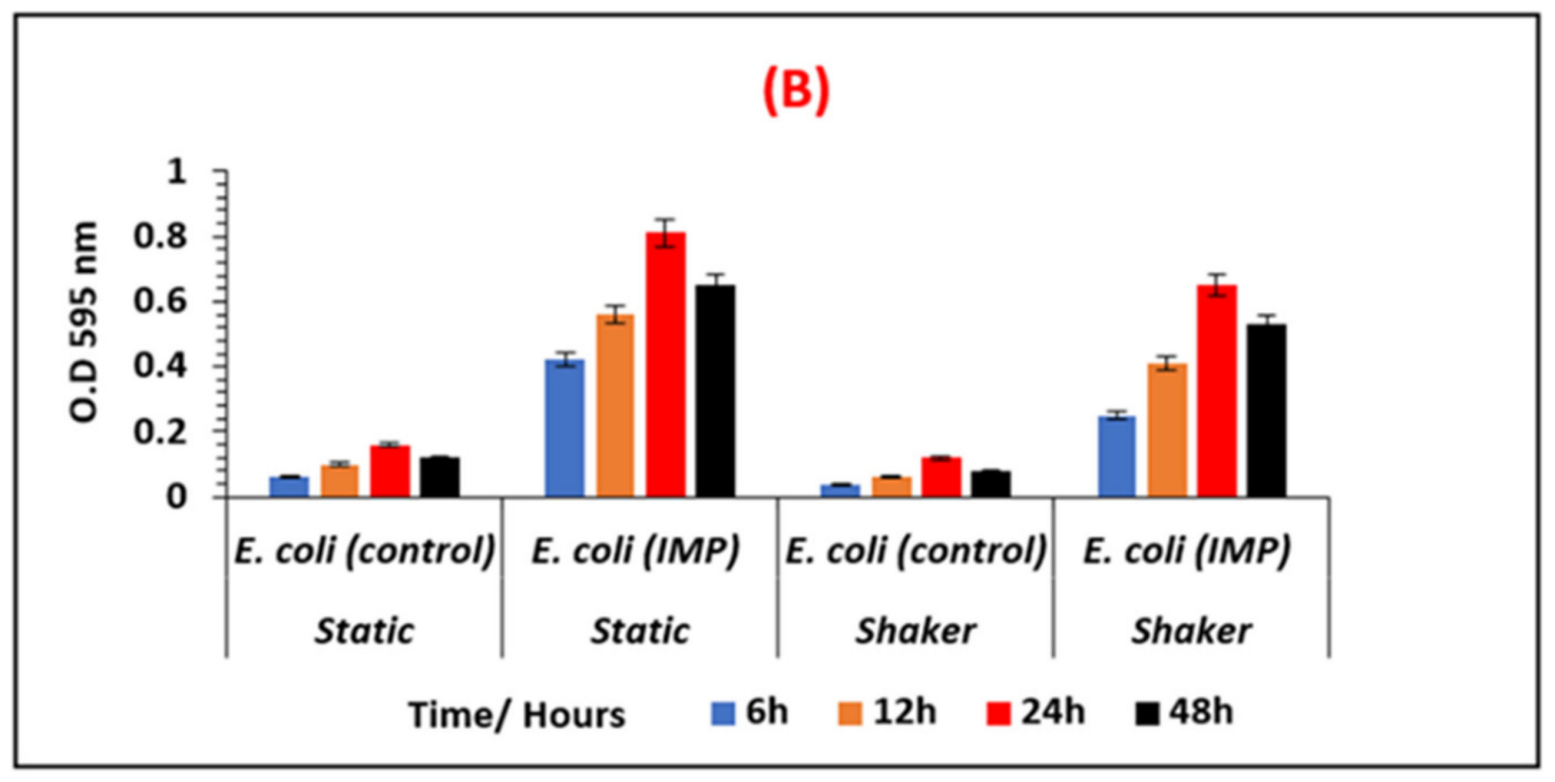
Comparison of the varying biofilm levels formed by *E. coli* 12241 (control) and *E. coli* IMP (CRE strain). Biofilms are quantified and measured at OD _595_ nm, at different growth stages (6 h, 12 h, 24 h, and 48 h), under shaken and static conditions, in nutrient-poor AB media (**A**), and in nutrient-rich LB media (**B**).

**Figure 3:**
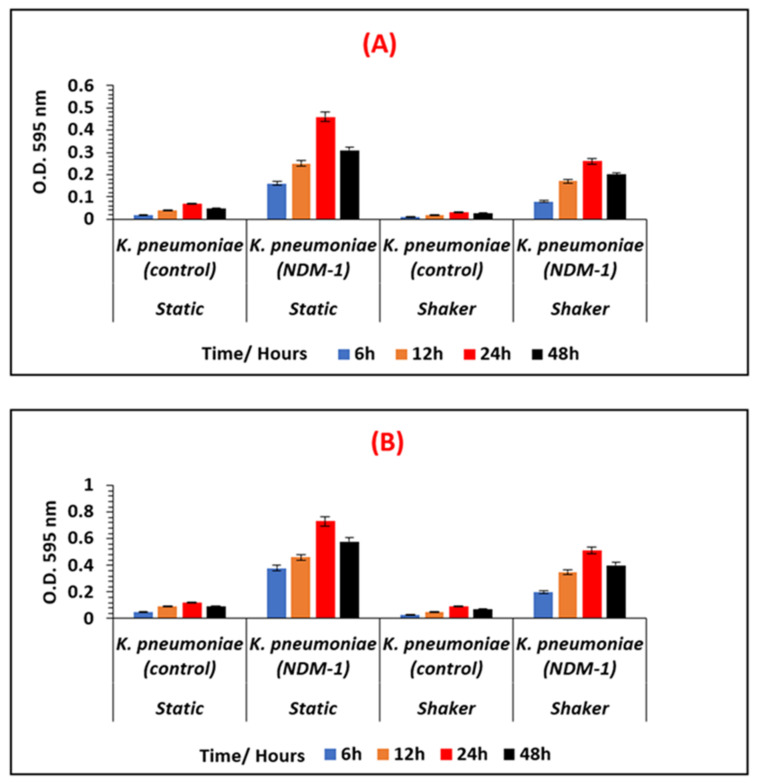
Comparison of the varying biofilm levels formed by *K. pneumoniae* 9633 (control), and *K. pneumoniae* NDM-1 (CRE strain). Biofilms are quantified and measured at OD _595_ nm, at different growth stages (6 h, 12 h, 24 h, and 48 h), under shaken and static conditions, in nutrient-poor AB media (**A**), and in nutrient-rich LB media (**B**).

**Figure 4:**
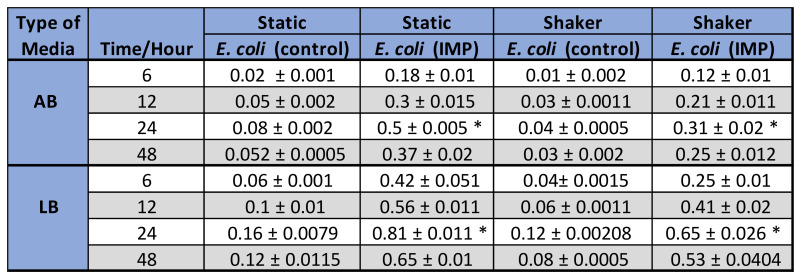
The values represent mean biofilm values (OD_595_) with standard deviations (SD) in non-resistant *E. coli* (12241) and CRE strain, *E. coli* IMP, growth in AB and LB media and under different growth conditions (static and shaker), and growth stages (6 h, 12 h, 24 h, and 48 h). A statistically significant difference in biofilm density between the four different time points is indicated as a star *. The target sample at 24 h is statistically significant compared to the other time-points at 6 h, 12 h, and 48 h.

**Figure 5:**
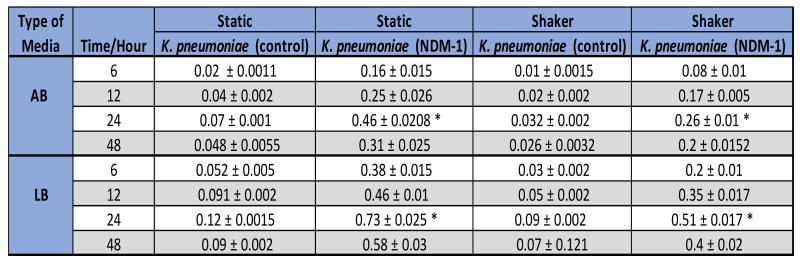
The values represent mean biofilm values (OD_595_) with standard deviations (SD) in non-resistant control *K. pneumoniae* 9633, and CRE strain *K. pneumonia* NDM-1 in AB and LB media, under different growth conditions (static and shaker), and growth stage (6, 12, 24, and 48 h). A statistically significant difference in biofilm density between the four different time points is indicated as a star *. The target sample at 24 h is statistically significant compared to the other time-points at 6 h, 12 h, and 48 h.

**Figure 6:**
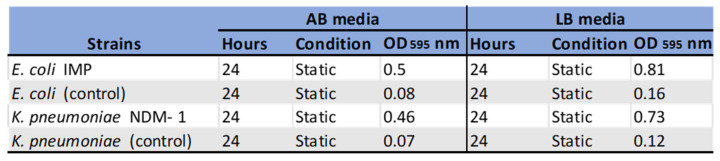
Compression of adherence levels and maximum amount of biofilm formation in non-resistant and resistant strains under the growth conditions tested. Biofilms were classified in categories as strongly adherent (OD_595_ > 0.5), moderately adherent (OD_595_ 0.3–0.5), weakly adherent (OD_595_ < 0.3), Lack biofilm OD_595_ < 0.15, and non-adherent (OD_595_ = 0).

The varying amounts of biofilm formed by the non-resistant control, *E. coli* 12241 and the CRE strain *E. coli* IMP at different growth conditions is represented in Figure 2A,B respectively. Similarly, Figure 3A,B represent the biofilm quantities formed by the non-resistant control *K. pneumoniae* 9633 and CRE stain *K. pneumonia* NDM-1. Figure 4 and Figure 5 show the mean values of the quantified biofilms including the statistical significance. Figure 6 compares the various adherence levels of non-resistant and resistant strains under the growth conditions tested.

As shown in Figure 2, a high amount of biofilm formation was observed for antibiotic-resistant *E. coli* IMP compared to non-resistant *E. coli* 12241 control. As indicated in Figure 6 *E. coli* IMP was strongly adherent with values of OD_595_ > 0.81 compared to the non-resistant control *E. coli* strain, with its weak adherence of OD_595_ < 0.16. For both non-resistant and resistant *E. coli* strains, varying amounts of biofilm formation were observed corresponding to the biofilm development stages, where statistically significant (*p* < 0.05), maximum biofilm formation was observed at the 24-h incubation period under all the conditions tested (Figure 4). *E. coli* IMP showed strong adherence at the 24 h, and under static conditions in AB with an OD_595_ of 0.5, and LB OD_595_ of 0.81, respectively (Figure 4).

According to Figure 3, a similar pattern of biofilm development was observed for *K. pneumoniae* strains, where a greater amount of biofilm formation was observed for the CRE strain *K. pneumoniae* NDM-1 compared to the non-resistant *K. pneumoniae* 9633 (control) under all conditions tested. As indicated in Figure 6, *K. pneumoniae* NDM-1 was strongly adherent with an OD_595_ > 0.73 compared to the non-resistant control *K. pneumoniae* strain with a weak adherence of OD_595_ < 0.12. For both *K. pneumoniae* strains, varying amounts of biofilm formation were observed corresponding to the biofilm development stages, where statistically significant (*p* < 0.05), maximum biofilm formation was observed at the 24-h incubation period under all the conditions tested (Figure 5). *K. pneumoniae* NDM-1 also showed strong adherence at the 24 h, and under a static condition with an OD_595_ of 0.46 in AB media, and an OD_595_ of 0.73 in LB media, respectively (Figure 5).

### 3.2. Confocal Laser Scanning Microscopy (CLSM)

**Figure 7:**
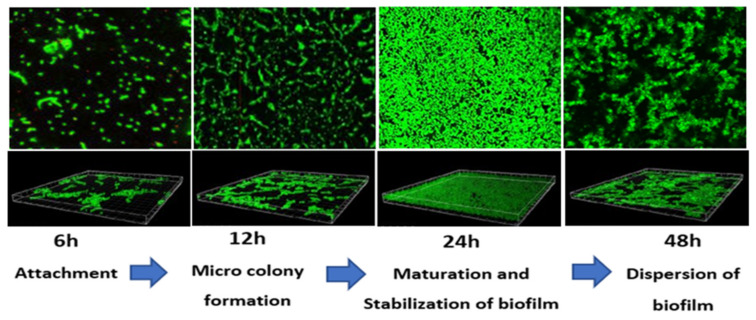
Representative CLSM images of *E. coli* IMP in different stages of the biofilm development. Live cells fluoresce in green with Syto 9 dye and dead cells are stained red with propidium iodide (PI). Original magnification: X200 and dimension; length 1.5 × width 1.5 mm. Lower panel represents the optical thickness obtained at each different biofilm growth stage.

Figure 7 shows the morphological observation of different biofilm growth stages in *E. coli* IMP. According to Figure 7, the cell surface-associated meshwork-like structures are weak at 6 h but showed a moderate form of the structures at 12 h. Around 24 h, the meshwork structure becomes denser, whilst dispersion of these structures was observed at 48 h. Under the above observation, the average optical thicknesses were determined as 0.3 µm, 0.55 µm, 1.7 µm, and 0.9 µm, corresponding to each time point at 6 h, 12 h, 24 h, and 48 h, respectively (Figure 7 lower panels).

**Figure 8:**
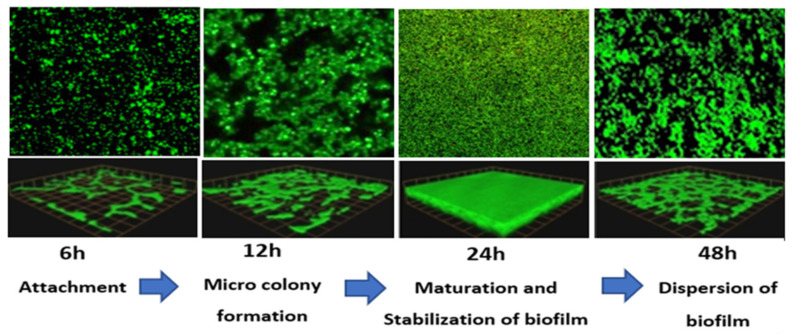
Representative CLSM images of *K. pneumoniae* NDM-1 in different stages of the biofilm development. Live cells fluoresce in green with Syto 9 dye and dead cells are stained red with propidium iodide (PI). Original magnification: X200 and dimension; length 1.5 × width 1.5 mm. Lower panel represents the optical thickness obtained at each different biofilm growth stage.

Similarly, *K. pneumoniae* NDM-1 showed that cell surface-associated meshwork like structures is weak at 6 h, with the moderate formation of structures at 12 h. Around 24 h the meshwork structure becomes denser, with a dispersion of these structures observed at 48 h (Figure 8). Additionally, for *K. pneumoniae* NDM-1, the average optical thicknesses of 0.2 µm, 0.5 µm, 1.5 µm, and 1.1 µm, were observed corresponding to each time point at 6 h, 12 h, 24 h, and 48 h, respectively (Figure 8 lower panels). Interestingly, under all the growth conditions tested, the majority of morphological structures appeared live (green colour) in both *E. coli* IMP and *K. pneumoniae* NDM-1, even though they underwent the chemical fixation process during biofilm analysis.

### 3.3. Stability of RNA, Selection of Reference Genes, Primer Efficiencies, Specificity of Primers for qPCR

The quality and quantity of RNA extracted at different time points and growth conditions for both strains were found to be satisfactory. The most stable reference (housekeeping) genes from a set of selected candidates were determined by using the geNorm algorithm (https://genorm.cmgg.be/ (accessed on 12 December 2019)). From this, a gene expression normalisation factor was calculated for each target gene based on the geometric mean of a selected number of genes, according to the previously described method [33,34]. For *E. coli* IMP, *16SRNA*, *rssA*, and *galE* were selected as reference genes and, according to geNorm, *16sRNA* and *rssA* produced the lowest M-values (0.082) in combination, indicating they were the most stably expressed genes across all samples. Similarly, for *K. pneumoniae* NDM-*1 16SRNA*, *recA* and *tonB* were selected as reference candidates, and according to geNorm, *16S RNA* and *recA* produced the lowest M-values (0.21) in combination, indicating they were the most stably expressed genes across all samples.

The primer efficiencies for genes tested for both *E. coli* IMP and *K. pneumoniae* NDM-1 were evaluated using calculated serial dilutions of cDNA (100 ng/µL). The primer efficiency of 90–110% was accepted in the qPCR analysis, and the required efficiency was observed for all the primers to the target genes (Figure 1).

**Figure 9:**
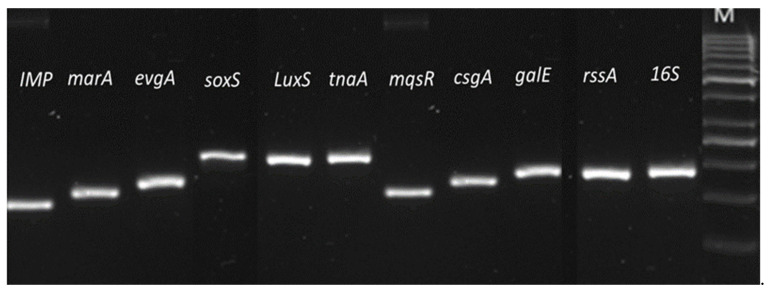
The specificity of each primer set for *E. coli* IMP gene targets. End amplified products of qPCR showing all primer pairs resulted in a single band on an agarose gel. M indicates the DNA ladder used, Gene Ruler 50 bp DNA Ladder. Products and ladder ran on 1% Agarose gel.

**Figure 10:**
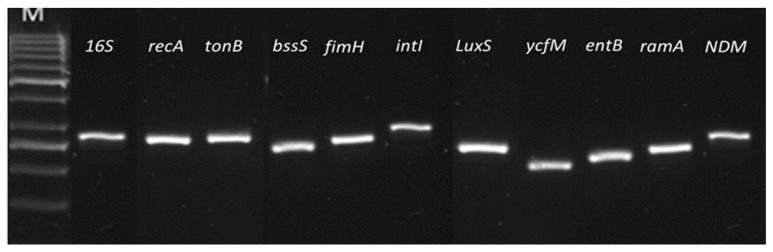
The specificity of each primer set for *K. pneumoniae* NDM-1 gene targets. End amplified products of qPCR showing all primer pairs resulted in a single band on an agarose gel. M indicates the DNA ladder used, Gene Ruler 50 bp DNA Ladder. Products and ladder ran on 1% Agarose gel.

Melting curve analysis was used at the end of qPCR products amplification to determine the specificity of each primer set for target genes in *E. coli* IMP and *K. pneumoniae* NDM-1. For both strains, it produced one peak and one product for each tested primer pair. The endpoint qPCR products visualised by agarose gel electrophoresis showed that all primer pairs resulted in amplification of a single product of the selected target gene (see Figure 9 and Figure 10 for *E. coli* IMP and *K. pneumoniae* NDM-1, respectively).

### 3.4. Expression Levels of Selected Antibiotic-Resistant and Biofilm-Associated Genes in E. coli IMP

**Figure 11:**
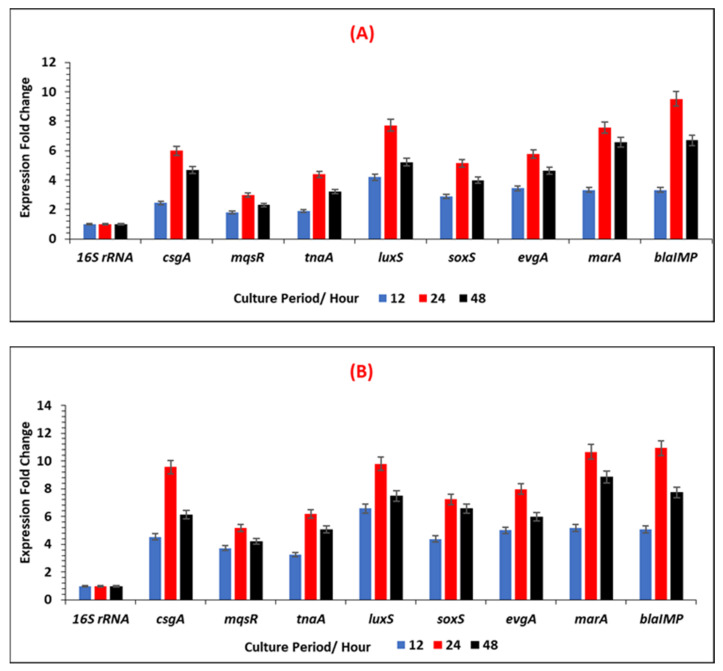
The expression fold change of each selected gene for biofilm-maturation, quorum sensing, stress survival, and antibiotic-resistant in ***E. coli* IMP** under different growth conditions in (**A**) nutrient-poor AB media, and (**B**) in nutrient-rich LB media. Fold change was calculated considering a calibrator sample at a 6 h period and normalised to the expression of reference gene *16S RNA*.

**Figure 12:**
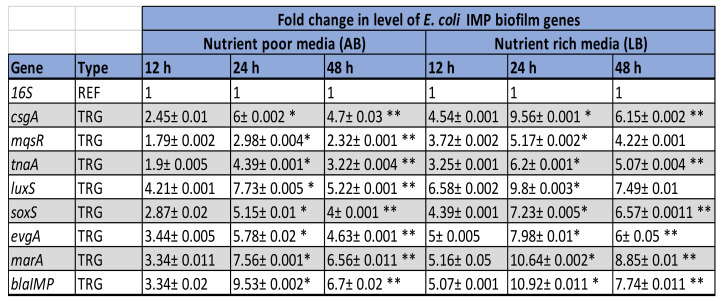
The expression fold change of Antibiotic-resistant and biofilm-related genes in ***E. coli* IMP** grown in AB and LB broth at different time points of 12, 24, and 48 h. TRG represents the target gene and REF indicates the reference gene (REF)]. The statistically significant increased fold expression value is considered when *p*-value < 0.05 and indicated as *. The statistically significant decreased fold expression value is considered when *p*-value > 0.05 and indicated as **.

Figure 11A,B and Figure 12 represents the relative changes in the expression levels of the selected gens of *E. coli* IMP, at different time points (12 h, 24 h and 48 h) of biofilm formation, in the two different growth media and indicates as fold expression values. A fluctuation of gene expression levels was observed over time, where initial upregulation was at the early adherence phase (12 h), and maximum upregulation at the mid-adherence phase (24 h), followed by a slight downregulation at the biofilm dispersion phase (48 h). This pattern of gene regulation was observed in both growth media, and all the gene categories (biofilms, quorum sensing, stress survival, and antibiotic-resistant genes), in *E. coli* IMP (Figure 1). In all genes tested, in both media, quorum sensing genes, such as *luxS*, were highly upregulated at 24 h; similarly, the highest upregulation of the antibiotic-resistant genes (*marA* and *bla_IMP_*) were also observed at 24 h. All the categories of genes were highly upregulated in the nutritionally nutrient-rich LB media compared to the nutrient-poor AB media.

According to Figure 12, all the target genes showed statistically significant increased fold expression values at 24 h and decreased at 48 h time point under the condition tested.

### 3.5. Expression Levels of Selected Antibiotic-Resistant and Biofilm-Associated Genes in K. pneumoniae NDM-1

**Figure 13:**
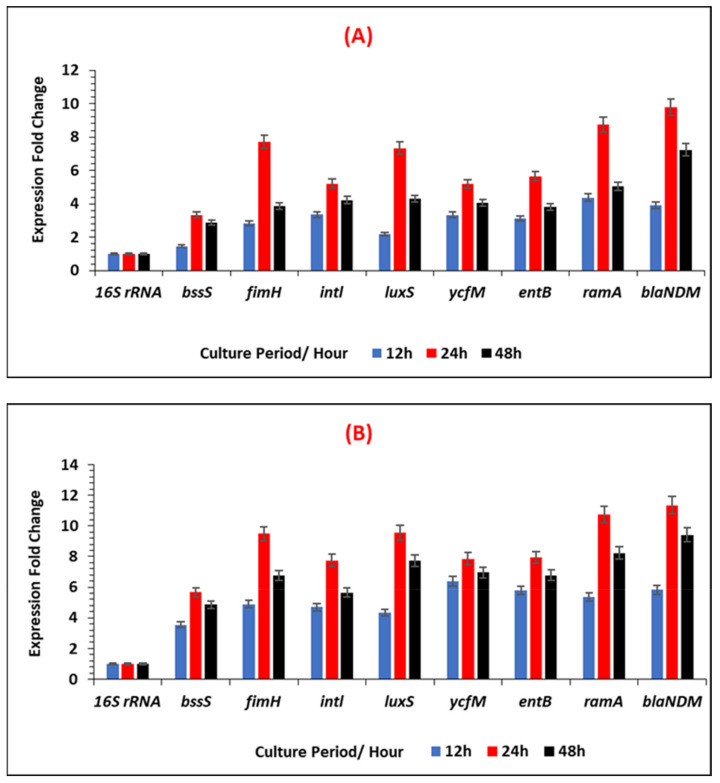
The expression fold change of each selected gene for biofilm-maturation, quorum sensing, stress survival, and antibiotic-resistant in ***K. pneumoniae* NDM** under different growth conditions in (**A**) nutrient-poor AB media, and (**B**) nutrient-rich LB media. Fold change was calculated considering a calibrator sample at 6 h time period and normalised to the expression of reference gene *16S RNA*.

**Figure 14:**
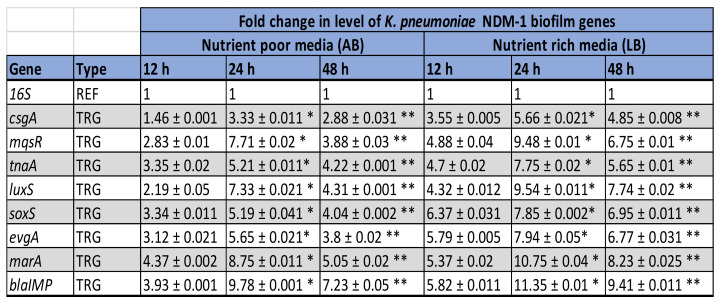
The expression fold change of Antibiotic-resistant and biofilm-related genes in *K. pneumoniae* NDM-1 grown in AB and LB broth at different time points of 12, 24, and 48 h. TRG represents the target gene and REF indicates the reference gene (REF)]. The statistically significant increased fold expression value is considered when *p*-value < 0.05 and indicated as *. The statistically significant decreased fold expression value is considered when *p*-value > 0.05 and indicated as **.

The relative changes in the expression levels of the selected genes at each different time point (12 h, 24 h, and 48 h) of biofilm formation, and in the two different growth media are represented as fold expression values for *K. pneumoniae* NDM-1 and are represented in Figure 13 and Figure 14. Similar to *E. coli* IMP, a fluctuation of gene expression levels was observed over time in *K. pneumoniae* NDM-1. An initial upregulation was observed in the early adherence phase (12 h), and maximum upregulation at the mid-adherence phase (24 h), followed by a slight downregulation at the biofilm dispersion phase (48 h). This pattern of gene regulation was observed in both growth media, and for all the gene categories (biofilms, quorum sensing, stress survival, and antibiotic resistance genes; Figure 1). In all genes tested, in both media, quorum sensing genes such as *intl* and *luxS* were highly upregulated at 24 h. Similarly, the highest upregulation of the antibiotic resistance gene, *bla_NDM_*, was also observed at 24 h. All the categories of genes were highly upregulated in *K. pneumoniae* NDM-1 in the nutritionally rich LB media compared to the nutrient-poor AB media, in a similar manner to *E. coli* IMP (Figure 12).

## 4. Discussion

Biofilm formation is a significant characteristic of Enterobacterales including *Escherichia coli* and *Klebsiella pneumoniae* [35]. Growth in biofilms promotes the survival of bacterial inhabitants in hospital settings and internal patients, rising the probability of occasional nosocomial infections [36,37,38]. Multidrug-resistant CRE, such as *E. coli* IMP and *K. pneumoniae* NDM-1, have been reported as a significant health challenge as they are associated with common community and hospital-acquired infections, and indeed CRE outbreaks have already been reported worldwide [21,22,23,24,25,26,27,28,29,30,31,32,33,34,35,36,37,38,39]. The clinical risk factors for bacterial infections due to CRE were reported as being well captured; however, bacterial risk factors such as antibiotic susceptibility, resistant mechanisms, and biofilm formation, for those infections due to CRE are limited. Hence, understanding the bacterial characteristics, such as biofilms is of utmost importance to the treatment of the biofilm-associated infections caused by CRE pathogens due to the associated poor clinical outcomes and limited therapeutic options associated with these pathogens.

Here, we observed the potential relationship between biofilm formation and the existence of antimicrobial resistance genes. Strong biofilm formation was observed for both *E. coli* IMP and *K. pneumoniae* NDM-1, which carry the *bla_IMP_* and *bla_NDM-1_* resistance genes, compared to the non-resistant control strains. This rate of stronger biofilm formation was similarly observed in other studies of multidrug-resistant *Enterobacteriaceae* [40]. In addition to strong biofilm formation in *E. coli* IMP and *K. pneumoniae* NDM-1, the greatest upregulation of the resistance genes, *bla _IMP_* and *bla _NDM-1_* at 24 h, where optimal biofilm formation was observed. This could be due to the fact the ability of these resistance genes to influence the process of biofilm formation, and that has been demonstrated that resistance genes in special plasmids can regulate biofilm formation in *Klebsiella pneumoniae* [28].

The effect of aeration and distribution of nutrients on the biofilm formation process were also explored in this study by incubating test strains under shaking and static conditions. Our data revealed that both CRE strains were high biofilm formers when grown under static incubation as indicated in Figure 5, and that characteristic could be attributed to their preferred physiological growth condition of facultative anaerobes [41].

Both CRE strains’ biofilm analysis and CLSM revealed a varying amounts of biofilms correlating with the biofilm development stages, where the 6 h stage corresponded to attachment, the 12 h stage to exopolysaccharides secretion and microcolony formation, and the 24 h stage to maturation and stabilisation of a three- dimensional biofilm structure, with detachment and dispersion on the biofilm observed at the 48 h time point. This biofilm development was in accordance with the previously demonstrated studies on Gram-negative bacteria [42,43]. Moreover, both CRE strains demonstrated the maximum amount of biofilm at the biofilm maturation stage (24 h of incubation), as similarly observed in multidrug-resistant *P. aeruginosa* isolates [40]) and drug-resistant *E. coli* isolates [24].

In *E. coli* IMP, biofilm-related genes *csgA* and *mqsR* showed the highest upregulation at the biofilm maturation stage (24 h) to their role in the mechanism of biofilm formation [44,45]. *csgA* is part of the curli-specific *csgBAC* operon, which plays a role in curli-dependent biofilm development via encoding the protein Curli [44]. Their role of Curli in surface attachment and cell-to-cell contact makes them the main part of the biofilm matrices and a contributing factor to the biofilm development mechanism, for which it has been demonstrated that infection-associated bacteria are capable of developing curli-dependent biofilm structures [46,47,48]. Upregulation of the *mqsR* gene at the biofilm maturation stage could be attributed to its function in motility and biofilm formation, as has previously been described for *E. coli* [45].

For *K. pneumoniae* NDM-1, biofilm-related genes *bssS*, and also *fimH*, showed the highest upregulation at the biofilm maturation stage (24 h) to their role in the mechanism of biofilm formation [49,50]. *bssS* encodes a small cytoplasmic protein that is a significant regulator in biofilm formation via signal secretion [50]. *fimH* encodes for one of the most important virulence factors, fimbriae, which is important for attachment and adhesion and is also required for colonisation and biofilm formation [51].

The upregulation of the stress-responsive gene *soxS* in *E. coli* IMP is attributed to its response to the growth condition-related role in protecting bacteria from oxidative stress [23]. The presence of nitric oxide or superoxide-generating factors in growth conditions, such as controlled by locus *soxRS. soxR* senses the intracellular redox signals of these factors and acts as a potent activator of *soxS* transcription when being activated [52]. The *evgA,* another stress-responsive gene, encodes for proteins involved in bacterial signalling pathways that allow for a diversity of adaptive responses to their environment. These adaptive responses are often interceded by two-component regulatory systems which generally depend on a prospector kinase and a response regulator [53]. Collectively, both *soxS* and *evgA* play integral roles in biofilm development stages by adapting to different environmental stress responses and contributing to the virulence fitness of *E. coli* [54,55].

In *K. pneumoniae* NDM-1 the upregulating stress-responsive *entB* and *ycfM* could be implicated in the role of iron uptake in different growth/iron starvation conditions. This behaviour of upregulation of *entB* and *ycfM* genes in response to iron starvation was also observed in other parallel studies in poor nutrient and nutrient-rich environments [56,57].

For *E. coli* IMP, we observed maximum upregulation of the QS genes, *tnaA* and *luxS*, at the maturation stage of the biofilm development (24 h), which were highly upregulated irrespective of the growth medium. This finding is coincident with the observation that *tnaA* and *luxS* expression influences the microorganisms’ adaption to diverse environmental conditions, and also influences the virulence of biofilm formation [17]. Interestingly we have observed the most extensive upregulation of *tnaA* is in nutrient-rich media (LB) which contradicts the previous findings on the induction of quorum-sensing genes in nutrient-poor conditions [58].

In *K. pneumoniae* NDM-1, we observed high upregulation of the QS genes, *intl* and *luxS*, at the biofilm maturation stage (24 h in all growth conditions tested), which is following a recent study in an extensive drug-resistant *K. pneumoniae* clinical isolate where *luxS* gene expression played a significant role in biofilm formation, polysaccharide production, and metabolic pathways, and was influenced by nutrient availability and other environmental conditions [59].

The high-level upregulation of *bla_IMP_*, encoding for carbapenem resistance, could be attributed to the hypervirulence/resistance capability that this strain process at the biofilm maturation stage, which could correlate with its successful dissemination in many common infections [60]. The upregulation of antibiotic-resistant gene *marA* can also be attributed to its role in transcription activation as a part of the *marA/soxS/rob* regulon, where it regulates more than 40 genes of the *E. coli* chromosome, resulting in different levels of resistance to a wide array of antibiotics and superoxide’s [61]. Moreover, *marA* promotes the outflux and inhibition of the influx of antibiotics, giving rise to multiple antibiotic-resistant phenotypes [62].

Similarly, for *K. pneumonia* NDM-1, there is the highest upregulation of the carbapenem-resistant *bla_NDM_* gene and transcription activator *ramA* at the mature biofilm development stage (24 h). The high-level upregulation of *bla_NDM-1_* can be attributed to the hypervirulence/resistance that this strain process shows at the biofilm maturation stage. Collectively, the variation in the antibiotic-resistant genes tested in these strains could be attributed to the fact of their multi-drug resistant ability, as these strains harbour varieties of resistant determinants in their resistant plasmids that can easily conjugate with other bacteria, resulting in broad resistance to antibiotics [63]. The *bla*_NDM_ gene is generally accepted by conjugative plasmids, resulting in multiple additional determinants and leading to multidrug resistance [64,65,66]. *ramA* code for *ramA* is an actual transcription manager in *K. pneumoniae*, which has been established to be significant to the bacterial response to antimicrobial challenges and wider the regulatory role in *K. pneumonia* during infection microbe-host or microbe-drug interactions [67,68,69,70].

In this study, we considered only a cluster of genes encoding for antibiotic resistance (*bla_IMP_ bla_NDM-1_*, *marA*, and *ramA)*, genes encoding for biofilm maturation (*csgA*, *mqsR*, *bssS*, and *fimH*), genes encoding stress response (*soxS*, *evgA*, entB, and ycfM), and QS gene *luxS*, and their expression during the biofilm development stages. The formation of the biofilm confined into a selected cluster of genes on two selected resistant bacteria is a limitation of our study. Although present findings from our study indicated the potential role of the subset of genes selected on the biofilm formation, but performing additional studies, such as correlating the biofilm magnitude with the selected resistant determinants in CRE to understand the physiological impact of the biofilms is caused by differentiating gene expression, would allow us to understand the role of resistant genes as indicators for biofilm formation. This may help to better understand the molecular mechanism of biofilm formation over time. Our results showed that in both CRE strains, the gene expression is correlated with the biofilm development stages. The highest upregulation of all the gene clusters selected was observed at the biofilm maturation stage (24 h time point) and down-regulation was observed at the detachment stage (48 h time point) under all growth conditions tested. Therefore, the most interesting point in this study is the morphological imaging by CLSM coupled to qPCR which allowed correlating morphological modifications to gene expression profile.

## 5. Conclusions

This study evaluated the detailed gene expression analysis considering a cluster of genes that linked with the biofilm development in carbapenem-resistant enteric Gram-negative bacilli, *E coli* IMP and *K pneumoniae* NDM-1 strains. We observed that genes encoding for biofilm development are upregulated during biofilm growth in *E coli* IMP and *K pneumoniae* NDM-1 strains. Our data showed that biofilm growth, among the studied variables, is the major factor influencing the variability of biofilm gene expression quantification assays. CLSM combined with the qPCR has demonstrated that phenotypic biofilm development is correlated with the expression profile of the selected cluster of genes of biofilm, quorum sensing, stress survival, and antibiotic resistance. Therefore, this morphological analysis combined with the gene expression during the biofilm growth can be used to study variation in the regulation of such genes in these significant CRE pathogens. Considering our data and the published literature, it is thought that these CRE strains, *E. coli* IMP and *K. pneumoniae* NDM-1, with high biofilm-forming capacity in many growing conditions, indicate their ability to develop resistance to antibiotics and which is considered to be the major risk in terms of successful dissemination and risk in terms of the emergence of multidrug resistance among these CRE pathogens.
